# Rapid Determination of Three Organic Acids in Polygonum Vivipari Rhizoma via One Marker by HPLC-UV at Equal Absorption Wavelength and Effervescence-Assisted Matrix Solid-Phase Dispersion

**DOI:** 10.1155/2023/5546053

**Published:** 2023-06-28

**Authors:** Zhengming Qian, Dingqiang Huang, Zhuobin He, Qinghui He, Guoying Tan, Qi Huang, Yikuo Sun, Wenqing Li

**Affiliations:** ^1^College of Medical Imaging Laboratory and Rehabilitation, Xiangnan University, Chenzhou 423000, China; ^2^Key Laboratory of State Administration of Traditional Chinese Medicine, Dongguan HEC Cordyceps R&D Co., Ltd., Dongguan 523850, Guangdong, China; ^3^Amway (China) R&D Co., Ltd., Guangzhou 510730, China

## Abstract

A rapid HPLC-UV method for the determination of three organic acids (neochlorogenic acid, chlorogenic acid, and cryptochlorogenic acid) in Polygoni Vivipari Rhizoma (PVR) by one marker was developed. The sample was prepared by effervescence-assisted matrix solid-phase dispersion (EA-MSPD). The separation of compounds was performed on a Poroshell column. The equal absorption wavelength was set as follows: 292 nm (0∼7 min) and 324 nm (7∼10 min). The analytical time including sample extraction and HPLC separation time was 12 min. The analytical method validation such as accuracy (recoveries 99.85%–106.29% and RSD < 2.9%), precision (RSD < 1.3%), reproducibility (RSD < 1.7%), and stability tests (RSD < 0.7% in 24 h) proved that the established HPLC method was suitable for determination of three organic acids in PVR. The contents of three analytes obtained by the external standard method with three markers and the equal absorption wavelength method with one marker were similar (RSD ≤ 2.0%). The developed method, which is rapid and reference compound saving, is an improved quality evaluation method of PVR.

## 1. Introduction

Polygoni Vivipari Rhizoma (PVR), also called “Zhuyaliao” in Chinese, is a famous Tibetan folk medicine. Its dried root is usually used in checking diarrhea and activating blood circulation to dissipate blood stasis [1]. According to the literature studies, phenolic compounds (organic acids and flavonoids) are the main active constituents responsible for the antioxidant and bacteriostatic activities [2–4]. Among the phenolic compounds, the chlorogenic acid series compounds such as neochlorogenic acid, chlorogenic acid, and cryptochlorogenic acid have been found to exhibit good pharmacological activities [5]. Furthermore, these three compounds are also the primary organic acids found in PVR [6]. Therefore, simultaneous determination of three organic acids is crucial for the quality evaluation of PVR due to their good bioactivities and high contents.

To date, several HPLC methods for the determination of the three organic acids were reported by the external standard method (ESM) based on three reference compounds applied [7–10]. The chlorogenic acid is cheap, which is about 14 dollars per 20 mg, while the prices of neochlorogenic acid and cryptochlorogenic acid are relatively more expensive, which are both about 140 dollars per 20 mg. In order to reduce the cost of PVR sample test and simplify the method, it is necessary to develop an analytical method for the determination of the three organic acids by one cheap reference compound (chlorogenic acid). The quantitative analysis of multicomponents by single marker (QAMS) method has been applied in herbal medicines [11–14]. However, the relative calibration factor (RCF) is necessary to be established, which increases the operational complexity and limits the wide application of QAMS. Therefore, developing an HPLC method for determination the three compounds by one reference compound without RCF is preferable. These three organic acids performed different UV absorptions at different wavelengths. The chlorogenic acid may have the equal UV absorption with two other compounds at certain wavelengths. It is the equal absorption waveslength (EAW) of chlorogenic acid with neochlorogenic acid or cryptochlorogenic acid. Hence, developing a HPLC-UV method at the EAWs could realize simultaneous determination of the three components by chlorogenic acid without RCF.

In addition, due to the complex matrix of PVR and similar structures of chlorogenic acid and two other organic acids, the reported HPLC-UV methods for the determination of three organic acids, including extraction and separation, are always time-consuming (more than 25 min) [7–10]. In order to develop a rapid HPLC method for determining the three organic acids in PVR, the rapid sample extraction and HPLC separation should be considered. Effervescence-assisted matrix solid-phase dispersion (EA-MSPD), a modified MSPD method, is proved to be a simple, fast, and effective extraction technique. It promotes the microextraction process by generation of carbon dioxide in situ from the effervescent mixture consisting of a carbon dioxide source and an acid component dissolved in water [15–17]. Hence, EA-MSPD is a potential rapid extraction method for extracting organic acid from PVR. On the other hand, the Poroshell column is a kind of rapid HPLC column [18–20], which can provide the rapid separation of organic acids in PVR.

In the present study, a rapid and reference compound saving HPLC-UV method for the determination of neochlorogenic acid, chlorogenic acid, and cryptochlorogenic acid via one cheap marker (chlorogenic acid) in EAW is developed. The developed HPLC-UV method was successfully applied in the determination of organic acids in ten batches of PVR samples.

## 2. Materials and Methods

### 2.1. Chemicals and Materials

Neochlorogenic acid (99.9%), chlorogenic acid (99.0%), and cryptochlorogenic acid (99.2%) were purchased from Yuanye Biotechnology Co., Ltd. (Shanghai, China). HPLC-grade methanol was bought from Energy Chemistry Co., Ltd. (Shanghai, China). HPLC-grade acetic acid was purchased from Sigma-Aldrich Trading Co., Ltd. (Shanghai, China). Analytical grade methanol, sodium dihydrogen phosphate, and sodium carbonate were bought from Xilong Scientific Co., Ltd. (Shantou, China). Oxalic acid was obtained from Sigma-Aldrich Trading Co., Ltd. (Shanghai, China). Citric acid was obtained from Chengdu Kelong Chemical Co., Ltd. (Sichuan, China).

10 batches of PVR samples were collected from Sichuan, Yunan, and Guizhou Provinces and authenticated as the dried root of Polygonum Viviparum by Dr. Zheng-Ming Qian. Voucher specimens were deposited at Key Laboratory of State Administration of Traditional Chinese Medicine, Dongguan, Guangdong. All crude samples were smashed into powder using a tube mill (IKA, Guangzhou, China) and passed over 50 meshes.

### 2.2. Preparation of Reference Compound Solutions

1.5 mg/mL neochlorogenic acid, chlorogenic acid, and cryptochlorogenic acid were dissolved in 50% methanol, respectively. Mixed reference compound solutions were prepared by mixing them and diluted to the intended concentration with 50% methanol. All solutions were stored at 4°C.

### 2.3. Preparation of Sample Solution

The sample solution was prepared by the EA-MSPD method. In order to obtain the good extraction efficiency, different extraction conditions (composition of effervescent mixture, ratio of sample and effervescent mixture, milling time, polarity, and volume of extraction solvent) were studied by the single-factor method. The contents of three organic acids were used to evaluate the extraction efficiency.

The sample powder (0.25 g) and the effervescent mixture (0.592 g sodium carbonate and 0.658 g oxalic acid, molar ratio about 10 : 13) were precisely weighed and milled with the Retsch MM400 ball milling instrument (Retsch, Shanghai, China) for 1 min to obtain the homogeneous mixture. The 0.4 g mixture was accurately weighed into a 50 mL centrifuged polypropylene tube, and 4 mL of 20% methanol was added. The effervescence occurred instantly and lasted about 30 s. When the process ended, the extraction solution was vortexed (5 s) and filtered through a 0.22 *μ*m membrane before HPLC injection.

### 2.4. UV Condition

Three reference compounds were dissolved with 10% methanol containing 0.1% acetic acid (the HPLC mobile phase) to 19 *μ*g/mL (neochlorogenic acid 19.08 *μ*g/mL, chlorogenic acid 19.09 *μ*g/mL, and cryptochlorogenic acid 19.07 *μ*g/mL). The ultraviolet spectra of the three analytes were obtained by scanning the three reference compound solutions from 200 nm to 400 nm with Agilent Cary 60 ultraviolet spectrophotometer (Agilent Technologies, USA). 10% methanol containing 0.1% acetic acid was used as blank.

### 2.5. HPLC Condition

An Agilent 1260 II Series HPLC system (Agilent Technologies, USA) was employed for the analysis. The separation of compounds was achieved on an Agilent Poroshell 120 EC-C18 column (50 × 4.6 mm, 2.7 *μ*m) (batch number: B18386) at a column temperature of 35°C and eluted with 10% methanol containing 0.1% acetic acid at a flow rate of 1.0 mL/min in the isocratic mode. The detection wavelength was set at 0∼7 min (292 nm and 2 nm) and 7–10 min (324 nm and 2 nm). The injection volume was 2 *μ*L.

### 2.6. Method Validation

The method validation, including linearity, limit of detection (LOD), limit of quantification (LOQ), precision, accuracy, repeatability, and stability tests, was carried out.

#### 2.6.1. Linearity, LOD, and LOQ

A series of concentrations of reference component solutions were prepared for the evaluation of linearity. The neochlorogenic acid (0.32 to 477.00 *μ*g/mL), chlorogenic acid (0.64 to 477.18 *μ*g/mL), and cryptochlorogenic acid (0.95 to 476.65 *μ*g/mL) were analyzed by HPLC. The standard curve was constructed by plotting the peak area (*y*) versus the concentrations of reference compounds (*x*). The LODs and LOQs were determined by reference components and recorded as the corresponding concentrations, which gave the signal-to-noise (S/N) ratios approximately 3 and 10, respectively.

#### 2.6.2. Precision and Repeatability

The intra- and interday assays were used to assess the precision of the developed method. The intraday precision was determined by analyzing the reference components solution six times within one day. The interday precision was determined by analyzing the reference components solution twice per day for three days. The relative standard deviation (RSD) was used as a measure of precision. The repeatability of the developed method was evaluated by six replicates of the PVR sample analysis. The samples were extracted as “2.3” and analyzed as “2.4.” The RSD of the contents was used as a measurement of repeatability.

#### 2.6.3. Recovery

A recovery test was used to evaluate the accuracy of the developed method. Known amounts of three organic acids were added to the PVR sample powder and then extracted and analyzed by the developed method. The PVR sample was analyzed six times. The recovery rates were calculated as 100% × (found amount − original amount)/spiked amount.

#### 2.6.4. Stability and Robustness

The stability was assessed by analyzing PVR sample solution five times within 24 hours. Variation was evaluated by the RSD. The robustness studies were carried out by analyzing the reference solution with the developed method with small changes in method parameters as follows: flow rate (1.0 ± 0.1 mL/min) and column temperature (35 ± 3°C). The developed method was also tested on 3 different Agilent Poroshell 120 EC-C18 columns (batch number: B15046, B18386, and B19476) and 2 different instruments (Agilent 1260 I and Agilent 1260 II). With chlorogenic acid as the reference compound, the RRT values and the content of neochlorogenic acid and cryptochlorogenic acid in PVR samples were calculated for evaluation. Furthermore, the resolution of the 3 target peaks in PVR sample solutions was also used to evaluate the robustness.

## 3. Results and Discussion

### 3.1. Optimization of EA-MSPD Extraction Conditions

In order to obtain the good EA-MSPD method, different extraction conditions (composition of effervescent mixture, ratio of sample and effervescent mixture, milling time, polarity, and volume of extraction solvent) were studied by the single-factor method. The contents of three organic acids were used to evaluate the extraction efficiency. Tukey's honestly significant difference test was carried out to compare the organic acid contents at different levels of the investigated parameter.

**Figure 1:**
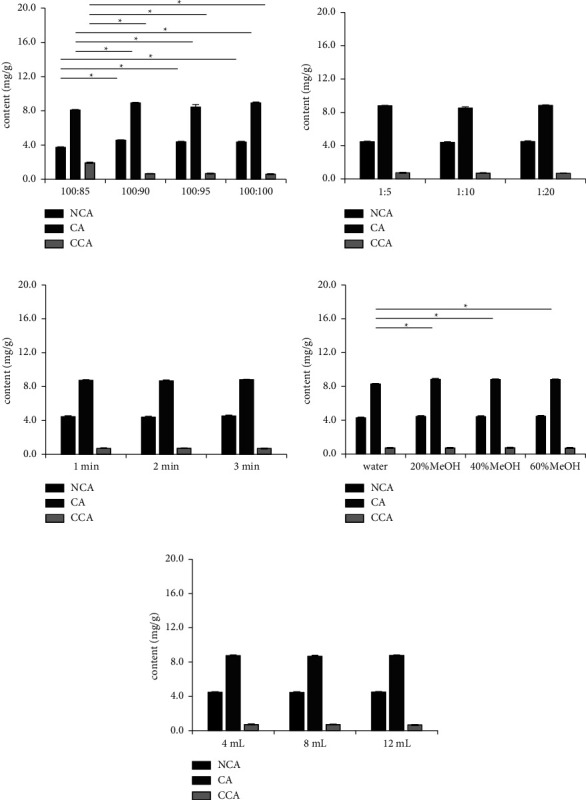
The extraction efficiency of three organic acids at different extract conditions. (a) Ratio of sodium carbonate to oxalic acid; (b) ratio of sample to effervescent mixture; (c) milling time; (d) concentration of methanol; and (e) volume of extraction solvent. NCA: neochlorogenic acid; CA: chlorogenic acid; and CCA: cryptochlorogenic acid. ^*∗*^*p* < 0.05 indicating significant difference, which was evaluated using Tukey's honestly significant difference test.

The effervescent mixture, a combination of the carbon dioxide source and acid component, had direct effect on the effervescent effect and extraction efficiency. Three commonly effervescent mixtures were tested, including sodium carbonate-oxalic acid, sodium carbonate-citric acid, and sodium carbonate-sodium dihydrogen phosphate [21–23]. It was observed that the effervescence effect of sodium carbonate-oxalic acid was more intense than two others, and the effervescence time (30 s) was faster than two others (more than 60 s). The ratio of sodium carbonate and oxalic acid and the ratio of sample and effervescent mixture were also important to the PVR sample extraction. According to the chemical reaction of sodium carbonate and oxalic acid, the molar ratio of sodium carbonate and oxalic acid is 1 : 1 (mass ratio of 100 : 85). The effervescent mixture (sodium carbonate: oxalic acid = 100 : 85) would make the sample solution in a weak alkaline environment. The organic acids are unstable in alkaline solution [24, 25]. So, more oxalic acid was added in the effervescent mixture to keep the sample solution in acid environment. Four different ratios of sodium carbonate and oxalic acid (100 : 85, 100 : 90, 100 : 95, and 100 : 100) were compared. As shown in Figure 1(a), the ratios of sodium carbonate and oxalic acid in 100 : 90, 100 : 95, and 100 : 100 showed better extraction efficiency. Stability tests also revealed that three organic acids were stable in 24 h at these ratios. Consequently, 100 : 90 was selected as the condition because of the less material cost. Three different ratios of sample and effervescent mixture (1 : 5, 1 : 10, and 1 : 20) were evaluated. As shown in Figure 1(b), the content of analytes was similar in the three conditions. The ratio of sample and effervescent mixture (1 : 5) was chosen for less material consume. After the composition of the effervescent mixture and the ratio of sample-effervescent mixture were fixed, the milling time (1, 2, and 3 min) was examined. The results (Figure 1(c)) of the three tests were similar, and 1.0 min was used in this study.

The polarity of the extract solvent would influence the solubility of the analytes. Methanol was selected as the extract solvent because of its wide practicability and superior capacity for extracting components from herbal medicine [4, 6–10, 26]. Different concentrations of methanol (0, 20, 40, and 60%) were compared. The results (Figure 1(d)) showed that 20%, 40%, and 60% methanol had better extraction efficiency than water. The 20% methanol was chosen based on the methanol cost. Different solvent volumes (4 mL, 8 mL, and 12 mL) were also examined.  Figure 1(e) reveals that 4 mL was sufficient to extract the analytes from PVR.

Compared with the votexing extraction method (non-EA-MSPD sample preparation), the extraction efficiency of the developed EA-MSPD method was improved 26.2% for neochlorogenic acid, 33.0% for chlorogenic acid, and 34.9% for cryptochlorogenic acid. To further confirm the extraction efficiency of the developed EA-MSPD, PVR sample S1 was extracted by the proposed EA-MSPD method and the reported ultrasonic extraction method [10], respectively. Three replicates were performed. The contents of neochlorogenic acid, chlorogenic acid, and cryptochlorogenic acid tested by the developed EA-MSPD method were 4.49 ± 0.01%, 8.88 ± 0.03%, and 0.76 ± 0.01% while the contents of these three analytes by the reported ultrasonic extraction method were 4.48 ± 0.05%, 8.59 ± 0.04%, and 0.71 ± 0.02%. These results show that the extraction efficiency of the developed EA-MSPD method is similar to that of the reported ultrasonic extraction method, which could be used for extracting three organic acids from PVR.

### 3.2. Optimization of HPLC Conditions

In order to develop a rapid HPLC separation of the three target compounds, the rapid HPLC column (Poroshell column) was employed. 0.1% acetic acid methanol based on the literature was used as the mobile phase system [10]. Three different mobile phases (8%, 10%, and 12% methanol with 0.1% acetic acid, respectively) were tested for separation. 10% methanol with 0.1% acetic acid was chosen as the eluting solvent for the good resolution and short separation time. The flow rate of 1.0 ml/min was used according to the literature [10]. Three different column temperatures (30, 35, and 40°C) were tested. The separations of analytes in three temperatures were similar, and 35°C was used in the current experiment as easy control and less energy consume.

**Figure 2:**
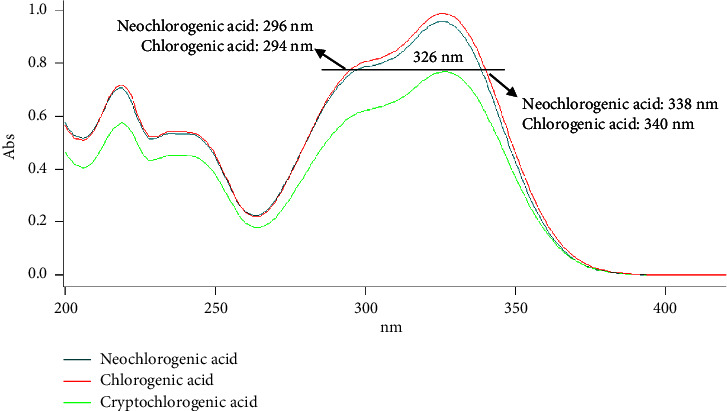
The UV spectrum of three organic acids.

Traditional QAMS often employs the maximum absorption wavelength of analytes, at which different compounds have different UV responses. So, the RCF is used for the determination of multiple compounds with one standard. In this study, three organic acids are detected at the EAW. The three analytes have the same response, and the RCFs are close to 1.0. So, it can test three compounds with one standard without RCF. Therefore, the selection of EAW is the key factor in the present HPLC method, which includes two steps (find and confirm EAW). First, screening the EAW by UV, three reference compound solutions at the same concentration were scanned from 200 nm to 400 nm with an ultraviolet spectrophotometer to get the UV spectrum for the three analytes. As shown in Figure 2, the UV response of cryptochlorogenic acid was lower than the other two compounds at the same UV wavelength. In order to obtain the better UV response of analytes, the maximum absorption wavelength (at 326 nm) was selected as the HPLC detection wavelength of cryptochlorogenic acid. The UV response of cryptochlorogenic acid (at 326 nm) was equal to noechlorogenic acid (at 296 nm and 338 nm) and chlorogenic acid (at 294 nm and 340 nm). Second, confirming the EAW by HPLC-UV, the mixed reference compound solution (neochlorogenic acid 63.60 *μ*g/mL, chlorogenic acid 63.62 *μ*g/mL, and cryptochlorogenic acid 63.55 *μ*g/mL) was injected to HPLC and detected at different wavelengths for confirming the EAW. The cryptochlorogenic acids were detected around 326 nm (±0 nm, ±1 nm, ±2 nm, and ±3 nm). It was found that cryptochlorogenic acid had the maximum peak area at 324 nm (Table S1). So, the detection wavelength of cryptochlorogenic acid was set at 324 nm. The noechlorogenic acid was detected around 296 nm (±0 nm, ±1 nm, ±2 nm, ±3 nm, and ±4 nm) and 338 nm (±0 nm, ±1 nm, ±2 nm, ±3 nm, and ±4 nm). The chlorogenic acid was detected around 294 nm (±0 nm, ±1 nm, ±2 nm, ±3 nm, and ±4 nm) and 340 nm (±0 nm, ±1 nm, ±2 nm, ±3 nm, and ±4 nm). The results (Table S1) showed that the peak areas of noechlorogenic acid (at 292 nm and 338 nm) and chlorogenic acid (at 292 nm and 339 nm) had the same peak areas with cryptochlorogenic acid (at 324 nm). Considering less detection wavelengths used, 292 nm was chosen as the detection wavelength for noechlorogenic acid and chlorogenic acid. In addition, the different bandwidths (1 nm, 2 nm, 4 nm, and 8 nm) were compared for the detection of three reference compounds at EAW. The results showed that the lowest RSD of peak areas could be obtained at 2 nm. The EAW conditions were as follows: 0∼7 min (292 nm, 2 nm) for detecting noechlorogenic acid and chlorogenic acid and 7–10 min (324 nm, 2 nm) for detecting cryptochlorogenic acid.

### 3.3. Method Validation

The validation of the current methods is summarized in Tables 1–4. The analytical method showed good linearity in the tested range with correlation coefficient *R* = 0.9999. The LODs and LOQs of the three analytes were less than 0.7 *μ*g/mL and 1.0 *μ*g/mL, respectively. The RSDs of intraday and interday precision were less than 1.3%. The RSDs of repeatability were less than 1.7%. The RSDs of stability were less than 0.7% within 24 hours. The recoveries of three analytes were 99.85∼106.29% (RSD less than 2.9%). In the robustness test, the RSDs of both contents and RRTs of noechlorogenic acid and cryptochlorogenic acid (determinated by chlorogenic acid) were all less than 2.0%. The resolutions of the 3 target peaks to the adjacent peaks were all larger than 1.5.

### 3.4. Analysis of Sample

**Figure 3:**
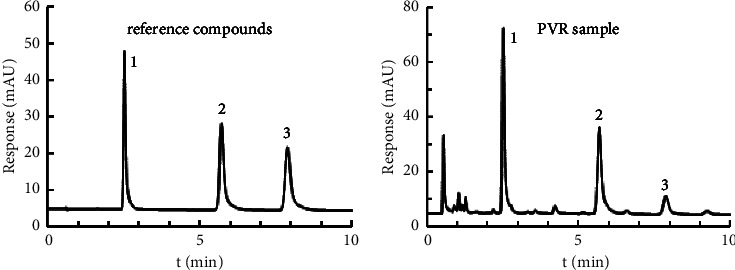
Chromatograms of reference compounds and PVR sample. (1) neochlorogenic acid; (2) chlorogenic acid; and (3) cryptochlorogenic acid.

The developed HPLC-UV EAW method was successfully applied in the determination of the target components in PVR samples. The chromatograms of the reference compounds and sample are shown in Figure 3, and the results are listed in Table 5. To confirm the feasibility of the developed HPLC-UV EAW method, the contents of three organic acids in ten PVR samples were determined by the ESM (with three reference compounds) and EAW method (with chlorogenic acid), respectively. The RSDs of the results obtained by the two methods were not more than 2.0%. These results indicated that the developed HPLC-UV EAW method could be used for quantitative analysis of three organic acids in PVR sample. The contents of noechlorogenic acid (1.24∼8.25 mg/g), chlorogenic acid (1.75∼13.75 mg/g), and crptochlorogenic acid (0.65∼2.10 mg/g) in PVR samples were agreed with the literature data [10].

### 3.5. Comparisons of the Developed and Previously Reported Methods

Several HPLC methods for analyzing these three organic acids have been reported [7–10]. Compared with these reported methods, the developed method is reference compound saving, simple, and fast.

The reported methods employed ESM with three reference compounds applied. In addition, the traditional QAMS for the determination of the three target analytes with one marker, often performed at the maximum absorption wavelength 330 nm, at which neochlorogenic acid, chlorogenic acid, and cryptochlorogenic acid respond differently, resulted in which the RCFs of neochlorogenic acid and cryptochlorogenic acid to chlorogenic acid are required. In this study, the EAW method uses only chlorgenic acid for determination of three target analytes. No RCF is applied because EAW is employed, at which neochlorogenic acid, chlorogenic acid, and cryptochlorogenic acid have the same UV response, and RCFs are close to 1.0. So, the developed HPLC-UV EAW method was much simpler than the traditional QAMS method.

The literature's methods for analyzing the three organic acids consume more than 25 min. For example, the HPLC method developed by Haghi et al. [7] costs 120 min in sample extraction and takes 35 min in HPLC separation with a total time of 155 min. Another HPLC method developed by Honda et al. [9] consumes 60 min including sample extraction (30 min) and HPLC separation (30 min). In the current method, EA-MSPD is applied in the PVR sample extraction and the Poroshell column is executed in HPLC separation. The whole process only costs 12 min in total, including about 2 min of sample preparation and 10 min of HPLC separation. It is faster compared to the reported methods [7–10].

## 4. Conclusions

In the present study, a rapid HPLC-UV EAW method for simultaneous determination of three organic acids in PVR samples by chlorogenic acid is established. Compared with the reported methods, the developed method is rapid, simple, and reference compound saving. It would be a good improved method for quality evaluation of the major organic acids in PVR samples.

## Figures and Tables

**Table 1:** The linearity, LODs, and LOQs of analytes.

Analytes	Calibration curves	*R*	Test range (*μ*g/mL)	LOD (*μ*g/mL)	LOQ (*μ*g/mL)
Neochlorogenic acid	*y* = 4.3597*x*−3.8990	0.9999	0.32∼477.00	0.16	0.32
Chlorogenic acid	*y* = 4.4316*x*−5.5305	0.9999	0.64∼477.18	0.32	0.64
Cryptochlorogenic acid	*y* = 4.4577*x*−2.9198	0.9999	0.95∼476.65	0.64	0.95

**Table 2:** Precision, repeatability, and stability of the analytes.

Analytes	Precision (RSD %)		Repeatability (RSD %, *n* = 6)	Stability (RSD %, 24 h)
	Intraday (*n* = 6)	Interday (*n* = 6)		
Neochlorogenic acid	0.36	0.85	0.63	0.27
Chlorogenic acid	0.56	0.54	0.58	0.38
Cryptochlorogenic acid	1.28	1.12	1.61	0.67

**Table 3:** The recoveries of the analytes.

Analytes	Sample	Original (mg)	Added (mg)	Found (mg)	Recovery (%)	Average recovery (%)	RSD (%)
Neochlorogenic acid	1	0.4542	0.2396	0.6976	101.55	100.99	2.69
	2	0.4530	0.2396	0.6889	98.44		
	3	0.4533	0.2396	0.6859	97.03		
	4	0.4551	0.2396	0.7041	103.90		
	5	0.4530	0.2396	0.6968	101.73		
	6	0.4529	0.2396	0.7004	103.29		

Chlorogenic acid	1	0.3956	0.2052	0.6026	100.90	99.85	2.87
	2	0.3945	0.2052	0.5937	97.06		
	3	0.3948	0.2052	0.5919	96.06		
	4	0.3964	0.2052	0.6072	102.75		
	5	0.3946	0.2052	0.5986	99.43		
	6	0.3944	0.2052	0.6055	102.89		

Cryptochlorogenic acid	1	0.1101	0.0631	0.1767	105.54	106.29	0.78
	2	0.1098	0.0631	0.1773	106.90		
	3	0.1099	0.0631	0.1763	105.17		
	4	0.1103	0.0631	0.1781	107.30		
	5	0.1098	0.0631	0.1768	106.15		
	6	0.1098	0.0631	0.1771	106.71		

**Table 4:** Robustness tests for the sample solution.

Parameters		RRTs^*∗*^		Content (mg/g)^*∗∗*^	
		Neochlorogenic acid	Cryptochlorogenic acid	Neochlorogenic acid	Cryptochlorogenic acid
Current method		0.438	1.397	4.63	0.78

Flow rate (mL/min)	0.9	0.439	1.394	4.55	0.77
	1.1	0.440	1.391	4.55	0.77

Column temperature (°C)	32	0.428	1.429	4.59	0.77
	38	0.448	1.365	4.51	0.79

Column	B15046	0.434	1.375	4.56	0.76
	B19476	0.442	1.387	4.59	0.77

Instrument	Agilent 1260 I	0.442	1.385	4.60	0.77

^*∗*^RRT was calculated as the retention time of the tested compound/the retention time of chlorogenic acid. ^*∗∗*^Content was calculated based on the peak area of the tested compound/peak area of chlorogenic acid × concentration of chlorogenic acids.

**Table 5:** The content of three organic acids in PVR samples (*n* = 2).

No.	Source	Chlorogenic acid	Neochlorogenic acid			Cryptochlorogenic acid		
		ESM (mg/g)	EAW (mg/g)	ESM (mg/g)	RSD^*∗*^ (%)	EAW (mg/g)	ESM (mg/g)	RSD^*∗*^ (%)
S1	Sichuan	8.83 ± 0.04	4.46 ± 0.04	4.45 ± 0.03	0.50	0.77 ± 0.01	0.76 ± 0.02	1.99
S2	Yunan	7.09 ± 0.13	8.25 ± 0.14	8.21 ± 0.16	1.65	2.10 ± 0.00	2.11 ± 0.00	0.21
S3	Sichuan	6.40 ± 0.01	4.23 ± 0.01	4.22 ± 0.00	0.12	0.86 ± 0.00	0.85 ± 0.00	0.74
S4	Sichuan	4.86 ± 0.04	2.95 ± 0.04	2.92 ± 0.02	0.83	0.81 ± 0.01	0.80 ± 0.01	1.14
S5	Guizhou	5.50 ± 0.01	2.05 ± 0.01	2.01 ± 0.01	1.02	0.65 ± 0.01	0.64 ± 0.01	1.93
S6	Yunan	8.61 ± 0.01	1.24 ± 0.01	1.20 ± 0.00	2.00	0.71 ± 0.00	0.70 ± 0.00	0.91
S7	Guizhou	4.29 ± 0.01	2.27 ± 0.01	2.24 ± 0.01	0.89	0.75 ± 0.01	0.74 ± 0.01	1.04
S8	Yunan	1.75 ± 0.00	2.32 ± 0.00	2.29 ± 0.01	0.86	0.72 ± 0.00	0.71 ± 0.00	0.89
S9	Guizhou	6.08 ± 0.05	2.48 ± 0.05	2.45 ± 0.01	0.79	0.66 ± 0.00	0.65 ± 0.00	0.95
S10	Sichuan	13.75 ± 0.09	3.55 ± 0.09	3.53 ± 0.03	0.67	1.44 ± 0.01	1.43 ± 0.01	0.67

^*∗*^RSD was obtained from the tested contents by the EAW method (*n* = 2) and the ESM method (*n* = 2).

## Data Availability

The data used to support the findings of this study are included within the article and the supplementary information file.

## Supplementary Materials

Supplementary Materials Table S1. The peak areas of neochlorogenic acid (63.60 *μ*g/mL), chlorogenic acid (63.62 *μ*g/mL), and crptochlorogenic acid (63.55 *μ*g/mL) at different detection wavelengths.
