# Supplementary material for: Rapid Determination of Three Organic Acids in Polygonum Vivipari Rhizoma via One Marker by HPLC-UV at Equal Absorption Wavelength and Effervescence-Assisted Matrix Solid-Phase Dispersion
Source: Int J Anal Chem. 2023 Jun 28;2023:5546053. doi: 10.1155/2023/5546053 (PMC10322645; doi:10.1155/2023/5546053)
Supplement: Supplementary Materials — Table S1. The peak areas of neochlorogenic acid (63.60 μg/mL), chlorogenic acid (63.62 μg/mL), and crptochlorogenic acid (63.55 μg/mL) at different detection wavelengths. [file 5546053.f1.docx]

**Rapid Determination of Three Organic Acids in Polygonum Vivipari Rhizoma via One Marker by HPLC-UV at Equal Absorption Wavelength and Effervescence-assisted Matrix Solid-phase Dispersion**

Zhengming Qian ^1, 2^, Dingqiang Huang^3, *^, Zhuobin He ^2^, Qinghui He ^3^, Guoying Tan ^2^, Qi Huang ^2^, Yikuo Sun^2^, and Wenqing Li ^2, *^

^1^ College of Medical Imaging Laboratory and Rehabilitation, Xiangnan University, Chenzhou 423000, China

^2^ Key Laboratory of State Administration of Traditional Chinese Medicine, Dongguan HEC Cordyceps R&D Co. Ltd., Guangdong 523850, China

^3^ Amway (China) R&D Co. Ltd., Guangzhou 510730, China

* Correspondence: david.huang@amway.com (Dingqiang Huang); liwenqing61@163.com (Wenqing Li).

**Table S1** The peak areas of neochlorogenic acid (63.60 μg/mL), chlorogenic acid (63.62 μg/mL) and crptochlorogenic acid (63.55 μg/mL) at different detection wavelengths

**C**

**B**

**A**

| **Cryptochlorogenic acid** | |  | **Neochlorogenic acid** | | | |  | **Chlorogenic acid** | | | |
| --- | --- | --- | --- | --- | --- | --- | --- | --- | --- | --- | --- |
| **Detection**  **Wavelength**  **（326±3 nm）** | **Peak**  **area** |  | **Detection**  **Wavelength**  **（296±4 nm）** | **Peak**  **area** | **Detection**  **Wavelength**  **（338±4 nm）** | **Peak**  **area** |  | **Detection wavelength**  **（294±4 nm）** | **Peak**  **area** | **Detection wavelength**  **（340±4 nm）** | **Peak**  **area** |
| 323 nm | 248.05 |  | **292 nm** | **249.44** | 334 nm | 287.19 |  | 290 nm | 241.19 | 336 nm | 272.60 |
| **324 nm** | **248.95** |  | 293 nm | 253.36 | 335 nm | 278.02 |  | 291 nm | 243.28 | 337 nm | 264.54 |
| 325 nm | 248.57 |  | 294 nm | 259.31 | 336 nm | 272.11 |  | **292 nm** | **247.52** | 338 nm | 256.70 |
| 326 nm | 247.67 |  | 295 nm | 261.04 | 337 nm | 264.05 |  | 293 nm | 251.26 | **339 nm** | **247.60** |
| 327 nm | 246.37 |  | 296 nm | 263.38 | **338 nm** | **250.19** |  | 294 nm | 256.22 | 340 nm | 238.03 |
| 328 nm | 244.86 |  | 297 nm | 265.65 | 339 nm | 242.32 |  | 295 nm | 259.56 | 341 nm | 229.98 |
| 329 nm | 242.66 |  | 298 nm | 267.27 | 340 nm | 232.51 |  | 296 nm | 262.37 | 342 nm | 223.12 |
| / | / |  | 299 nm | 268.83 | 341 nm | 224.94 |  | 297 nm | 264.64 | 343 nm | 216.46 |
| / | / |  | 300 nm | 270.40 | 342 nm | 217.91 |  | 298 nm | 266.37 | 344 nm | 210.05 |
